# Isokinetic leg muscle strength relationship to dynamic balance reflects gymnast-specific differences in adolescent females

**DOI:** 10.3389/fphys.2022.1084019

**Published:** 2023-01-09

**Authors:** Oľga Kyselovičová, Erika Zemková, Katarína Péliová, Lenka Matejová

**Affiliations:** ^1^ Department of Gymnastics, Dance, Fitness & Combat Sports, Faculty of Physical Education and Sport, Comenius University in Bratislava, Bratislava, Slovakia; ^2^ Department of Biological and Medical Sciences, Faculty of Physical Education and Sport, Comenius University in Bratislava, Bratislava, Slovakia; ^3^ Sports Technology Institute, Faculty of Electrical Engineering and Information Technology, Slovak University of Technology, Bratislava, Slovakia; ^4^ Faculty of Health Sciences, University of Ss. Cyril and Methodius in Trnava, Bratislava, Slovakia; ^5^ Department of Physical Education and Sport, University of Economics Bratislava, Bratislava, Slovakia; ^6^ Department of Sports Diagnostics and Physiotherapy, National Sport Centre, Bratislava, Slovakia

**Keywords:** aerobic gymnasts, artistic gymnasts, biodex isokinetic leg strength test, rhythmic gymnasts, Y-balance test

## Abstract

Balance, together with other motor qualities, plays an important role in the successful execution of specific gymnastic skills. However, it is also not clear whether different demands on dynamic balance and power produced by lower limb can be observed in sport-specific differences among gymnasts of various modalities. The question also is as to what extent isokinetic leg muscle strength contributes to anterior and posterior postural stability in gymnasts. Therefore, the aim of the study was i) to compare variables of dynamic balance and isokinetic leg muscle strength in rhythmic, artistic, and aerobic gymnasts, and ii) to investigate the relationship of reach distances in anterior, posteromedial, and posterolateral directions, as well as the composite score in the Y-balance test with an isokinetic muscle strength during knee extension and flexion at different velocities in female gymnasts of various disciplines. Altogether seven aerobic, five artistic, and six rhythmic gymnasts performed the Y-balance test and isokinetic leg muscle strength test at 60°/s, 180°/s, and 300°/s. Results showed significant between-group differences in the composite score in the Y-balance test of the dominant (*F* = 3.536, *p* = .041) and non-dominant symmetry (*F* = 4.804, *p* = .015). Similarly, average power produced during knee extension and knee flexion at 60°/s, 180°/s and 300°/s differed significantly among these groups (all at *p*˂0.05). In addition, there was a significant relationship between the composite score of the dominant limb symmetry and isokinetic dominant limb extension strength at 60°/s (*r* = .54), 180°/s (*r* = .87), and 300°/s (*r* = .84) in aerobic gymnasts. The composite score of the dominant limb symmetry was also associated with isokinetic dominant limb extension strength, albeit only at 60°/s in both artistic (*r* = .60) and rhythmic gymnasts (*r* = .55). Such between-group differences may be ascribed to their different demands on maintenance of balance under dynamic conditions and leg muscle power within their sport specializations. Taking into account significant association between the dominant limb symmetry and isokinetic dominant limb extension strength, it may be concluded that both muscle strength and fast speeds contribute to dynamic balance in adolescent gymnasts.

## 1 Introduction

In gymnastics, as well as in other sport disciplines, there is a constant demand for an increasing performance level. Therefore, improvement involves the individualization based on an objective assessment of the level of the effort adaptation for each athlete, corresponding to the competition specific effort characteristics. High-performance gymnastics involves a high degree of physiological adaptation. Together with a complete development of motor skills conducting specific training sessions implies both knowing the specific skills determining high performance levels, and the methods of assessment and development of the specific abilities (Puiu and Dragomir, 2020; Mack et al., 2021). The evaluation of physiological and energetic demands during exercise of relatively short duration, changing intensity, and types of muscle activation is rather complicated. Additionally, in aesthetic sports technical parameters of the specific performance play an important role (Kyselovičová et al., 2016).

Maximal heart rate values measured during various gymnastic disciplines have mirrored technical demands of increasing difficulty. Currently, exercise heart rates exceed 190 beat. min^−1^ (Sawczyn et al., 2018; Urzeala et al., 2020) as compared to 135 to 151 beat. min^−1^ in the seventies (Jemni et al., 2001). Measurement of higher blood lactate values at the end of the routines in artistic (Marina and Rodríguez 2014; Arazi and Mehrtash, 2017), rhythmic (Gateva, 2014; Batista et al., 2018), and aerobic gymnastics (Kyselovičová and Danielová, 2012; Aleksandreviciene et al., 2015) suggest that anaerobic glycolysis has increased in importance. Glycolytic contributions differ between modalities. Data from energy cost studies demonstrate that gymnastics energy demands are bigger now (Jemni, 2017).

In comparison with frequently monitoring gymnastics intensity and load by means of heart rate and blood lactate a small number of research deals with motor abilities. Additionally, the success of each gymnast is highly dependent on their competitive level. Therefore, it is necessary to monitor improvement during the process of motor development of each athlete continuously (Lamosova et al., 2021) to allow the individualization of training loads (Russell et al., 1995; Zaporozhanov et al., 2015; Kochanowitz et al., 2017) as the mastery of the technique of individual elements depend on level of motor skills that ensure their implementation (Chernykh et al., 2021). The contribution of strength and power to gymnastic performance has increased during the last decades (Jemni et al., 2001) in order to successfully and safely perform a dynamic and diverse set of skills in sequence (Moeskops et al., 2019; Niespodzinski et al., 2021). Thus, a sport-specific testing is recommended. For instance, the evaluation of modified aerobic jumps reflects better sport-specific performance, and also training adaptation of aerobic gymnasts (Kyselovičová and Zemková, 2010a).

Balance and postural control are fundamental qualities in sports with variable and/or asymmetrical movements (Magaña et al., 2017), which involve various multidirectional patterns, including acceleration and/or deceleration, rapid directional changes, repetitive take-offs, and numerous jumps and landings (Hrysomallis, 2011; Pau et al., 2015), especially in female gymnasts (Opala-Berdzik et al., 2021). Cross-sectional studies revealed that gymnasts tended to have the best balance ability, followed by soccer players, swimmers, physically active subjects, and then basketball players (Hrysomallis, 2011; Mkaouer et al., 2017). Such findings are not surprising. Sánchez-Lastra et al. (2019) showed that children and adolescents could improve their balance skills within 5–10 weeks of proprioception-oriented gymnastic training. Thus, the observed higher proprioception performance in gymnasts should be, at least to some extent, related to the gymnastic training (Kochanowicz et al., 2015; Skopal et al., 2020; Busquets et al., 2021). Balance in gymnastics is considered one of the most important performance factors that correlate with selected drop jump parameters (Istenič et al., 2016). Even minimal loss of balance may affect the final score of the gymnast (FIG, 2021). Despite this, there are only few studies that deal with the impact of balance ability on success in performing complex gymnastic elements in routines (Bobo-Arce and Méndez-Rial, 2013). Balance impairment after exercise and its readjustments to pre-exercise level depends not only on intensity of proprioceptive stimulation but also on type of exercise (Kyselovičová and Zemková, 2010b). Moreover, post-exercise balance impairment under fatigue induced by jumps of different duration is not linearly related to the level of proprioceptive stimulation (Zemkova et al., 2010).

Most studies compared balance in gymnasts with the control group of non-athletes (Vuillerme et al., 2001; Aydin et al., 2002; Davlin, 2004; Asseman et al., 2008) or other athletes (Davlin, 2004; Bressel et al., 2007). Kochanowicz et al. (2018 & 2019) reported that different gymnastic apparatus led to the activation of specific muscles controlling balance, and that balance performance is closely related to lower extremity muscle strength. In addition, to avoid the injuries of lower limbs, it is strongly recommended to focus on the relationship between different types of intermuscular coordination and the occurrence of lower limb injuries, and to modify landing strategies (Niespodziński et al., 2021) requiring balance control. In a more recent study (Niespodziński et al., 2022), the authors observed the differences in proprioception between youth and adults. The children and adolescents aged 9–11 years were characterised by lower precision of elbow joint position and force senses, in comparison with adults. Thus, gymnastic training can possibly accelerate force sense development when higher loads are considered (Kochanowicz et al., 2017).

As previously reported, there are many scientific investigations dealing with different physiological and functional aspects of gymnastics. However, studies comparing several gymnastic disciplines, e.g.: Olympic (artistic and rhythmic gymnastics) and non-Olympic (aerobic gymnastics), are less frequent, even sporadic. Gymnastics is a unique sport that has experienced a continuous evolution over the years in terms of technique and execution, while preserving the aesthetic value of the exercise. Gymnastic routines involve many specific movements, as well as dance-based skill, and difficulty elements, with phenomenal technical and artistic requirements. Coordination, flexibility, precision, balance, and strength are important motor qualities (Veljković et al., 2018; Líška and Kremnický, 2022). Additionally, all gymnastic disciplines require precise spatial and temporal coordination of multi-joint limb movements with postural control (Leskovec & Pavletič, 2019). Despite these common features of gymnastic disciplines, however, there also exist certain specificities in each of them: artistic gymnastics demands performance of various elements, which are perfectly executed on the gymnastics apparatus; in rhythmic gymnastics, the greatest technical value is attributed to balances and flexibility while manipulating with various apparatus; aerobic gymnastics routines must demonstrate continuous movement patterns and perfectly executed difficulty elements without apparatus. Thus, during the routine, gymnasts perform a whole spectrum of complex movements defined by technical regulation and rulebooks–the Code of Points (FIG, 2021). Various jumps and acrobatic elements with precise take-offs and landings, turns, and dynamic balances are common demands in all gymnastic disciplines (Hall, et al., 2016; Abuwarda, 2020), which requires a combination of speed, strength, endurance, agility, and flexibility. Thus, those qualities are crucial parameters for training and performance. In addition, an optimal amount of leg volume and leg mass contribute to success in elite gymnasts (Basturg & Marangoz, 2018), and became an important performance parameter in gymnastics (Mohamed, 2011). Despite the benefits of strength training–such as better performance and fewer injuries–in general, strength is not regularly assessed. For the most part, only studies involving artistic and rhythmic gymnastics were found (Gasparetto et al., 2022). Studies also examined the relationship between lower extremity muscle strength and balance performance (Muehlbauer et al., 2015; Myers et al., 2018), as it is known that a developed dynamic balance performance in athletes has a protective effect in lower extremity injuries (Butler et al., 2014; O’Malley et al., 2014), and *vice versa*, lower extremity strength is quite important in displaying balance performance (Deniskina and Levik, 2001).

In the last decades, the isokinetic muscle contraction has become a popular method for evaluating dynamic muscle function. Ultimately, these measures are interpreted to represent dynamic muscle function, and are the basis of athletes screening (Drouin et al., 2004). The primary reason is the fact that the isokinetic dynamometers provide constant velocity with accommodation resistance throughout a joint’s range of motion and a more detailed evaluation of the torque generating capacity of the muscles involved in specific joint movements (Iossifidou et al., 2005; Schons et al., 2018). Moreover, these devices allow the calculation of muscle strength imbalance or asymmetry (Bamaç et al., 2008; Schons et al., 2018).

It is considered that there is a relationship between the knee circumference muscles’ strength which is the determinant in athletes’ performances and dynamic balance. There are studies in the literature evaluating the relation between lower extremity muscle strengths and balance performance (Mohammadi et al., 2012; Iri et al., 2016; Çelenk et al., 2018), and that increase in lower extremity isokinetic strength can be a key factor in decreasing the risk of injuries in athletes and preventing performance losses (Aka and Altundag, 2020). However, in the literature, the number of studies investigating the relationship between gymnasts’ isokinetic knee muscle strength and balance performance is insufficient. Even more, when considering the specific group of adolescents, the relationship between balance and knee muscle strength of various gymnastic disciplines has not been clearly stated.

Taking into the account the specificity of performance in these sports, differences in dynamic balance and isokinetic leg muscle strength in rhythmic, artistic, and aerobic gymnasts may be assumed. It may be also hypothesised that there exists: 1) a significant positive relationship between dynamic balance and endurance speed as well as fast speed in aerobic gymnasts, 2) a significant positive relationship between dynamic balance and fast speed in rhythmic gymnasts, and 3) a significant positive relationship between dynamic balance and fast speed in artistic gymnasts. Verification of these hypotheses was accomplished by i) comparing dynamic balance and isokinetic leg muscle strength variables in rhythmic, artistic, and aerobic gymnasts, and ii) investigating the relationship of reach distances in anterior, posteromedial, and posterolateral directions and the composite score in the Y-balance test with an isokinetic muscle strength during knee extension and flexion at different velocities in female adolescent athletes of rhythmic, artistic, and aerobic gymnastics.

## 2 Methods

### 2.1 Subjects

Three groups of elite rhythmic (*n* = 6), artistic (*n* = 5), and aerobic (*n* = 7) gymnasts volunteered to participate in the study (Table 1). Experimental subjects were not randomized into groups because this was deemed irrelevant to this study. Subjects were selected based on their long-term expertise and gymnastics specificity. In addition, they had practiced gymnastics since they were 5 years old, competed at the international level for the last 3 years, and were enrolled in a particular national team at the time of testing. All gymnasts were in the pre-competitive period and trained 20–24 h in five training sessions per week. The number of participants in each gymnastic group was therefore limited due to the selection of only elite gymnasts with such specific criteria. They were excluded if they have suffered from chronic ankle instability and lower limb musculoskeletal injury in the previous 6 months.

**TABLE 1:** Groups characteristics.

	Rhythmic gymnasts	Artistic gymnasts	Aerobic gymnasts	*p* values	F Values
	*n* = 6	*n* = 5	*n* = 7		
Age [years]	17.7 (.53)	14.4 (5.92)	15.87 (.73)	.00006	20.07
Height [cm]	165.00 (4.73)	156.0 (6.0)	167.14 (4.05)	.004	8.19
Body mass [kg]	55.37 (5.53)	48.38 (5.27)	60.20 (5.20)	.007	7.16
BMI [kg/m^2^]	20.3 (1.46)	19.92 (2.7)	21.54 (1.67)	.332	1.19
Skeletal muscle mass [kg]	25.78 (2.78)	22.92 (2.11)	26.89 (2.72)	.056	3.51
Skeletal muscle mass [%]	46.55 (1.10)	47.62 (1.38)	44.64 (1.68)	.181	1.86
Body fat mass [kg]	8.73 (1.49)	6.84 (2.13)	11.59 (1.75)	.001	10.82
Body fat mass [%]	15.78 (2.02)	15.08 (6.99)	19.23 (2.49)	.008	6.75
Body water [kg]	34.02 (3.36)	30.52 (2.61)	35.54 (3.23)	.045	3.84
Body water [%]	61.47 (1.47)	63.24 (2.41)	59.04 (1.80)	.006	7.48

Participants were required to attend a session at the National Sport Centre laboratory. All of them were informed of the main purpose and procedures of this investigation. Parents and gymnasts read the participant information leaflet and provided written consent prior to testing.

The procedures followed were in accordance with the ethical standards on human experimentation stated in compliance with the 1964 Helsinki Declaration and its later amendments. The project was approved by the ethics committee of the Faculty of Physical Education and Sport, Comenius University in Bratislava (No. 4/2022).

### 2.2 Study design

This study used a cross-sectional and correlational design in order to investigate between-group differences and the association of knee flexion and extension muscle strength with dynamic balance in elite female gymnasts. During the competitive season, each athlete visited the National Sport Centre laboratory, where dynamic balance by means of the Y-Balance Test was assessed and muscle strength variables during knee flexion and extension at different angular velocities (60°/s, 180°/s, and 300°/s) were measured using an isokinetic dynamometer.

To identify the participants’ own experienced leg dominance, the Waterloo Footedness Questionnaire was used (Elias et al., 1998). After completion of the questionnaire, body composition analysis was conducted using the InBody 770 bioelectrical impedance analyser (BioSpace, Korea). Body fat and lean mass were recorded and archived for torque normalization purposes.

**FIGURE 1:**
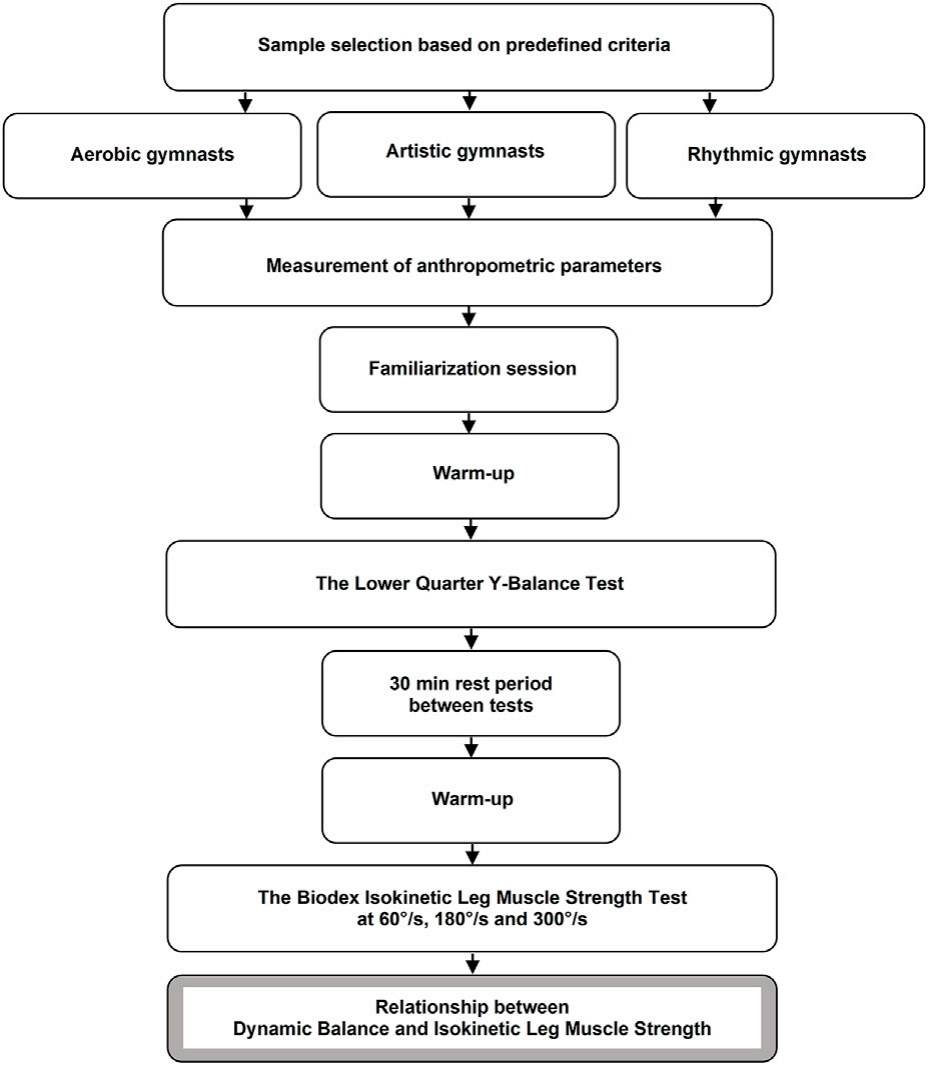
Flowchart of the experimental protocol.

Testing was divided in two phases with a 30 min rest interval in-between. The first phase started with 5 min running on a treadmill at a speed of 7 km per hour, followed by 10 min of foam rolling techniques and dynamic stretches. Then, an assessment of dynamic balance was conducted. Afterwards, the participants took a 30 min rest, before proceeding directly into the second phase. Prior to testing, they warmed-up by 5 min cycling on an ergometer with the resistance of 40–60 W, including a 15 s sprint at the beginning of the third and fourth min with the maximum frequency of revolutions (80–100 W). After the warm-up, the gymnasts’ isokinetic concentric strength of the dominant and non-dominant lower limb was assessed using the isokinetic dynamometer (Biodex Medical Systems Inc. NY, United States). All measurements took place in the morning, about 2 h after the first meal and in a state of good hydration. Schematic experimental protocol is shown in Figure 1.

### 2.3 Testing procedure

#### 2.3.1 The lower quarter Y-Balance test (YBT)

This test was used to assess dynamic balance. The Y-balance test has excellent inter-rater reliability for the anterior (ICC = .99; 95% CI: .99–1.0), posteromedial (ICC = 1.0; 95% CI: 1.0), and posterolateral (ICC = 1.0; 95% CI: .99–1.0) reaches and the composite score (ICC = 1.0; 95% CI: 1.0) (Alshehre et al., 2021).

YBT has been performed according to the previously described protocol by Plisky et al. (2009) and Gribble et al. (2012). The goal of this test was to maintain single-leg balance on one leg while reaching as far as possible with the contralateral leg in three different movement directions.

Prior to performing trials, participants received verbal instruction and visual demonstration of the YBT and were allowed two practice trials with corrective feedback (Robinson and Gribble, 2008). They started the test with the dominant lower limb**.** They were instructed to reach as far as possible in the anterior direction first, followed by the posteromedial and posterolateral directions, and to tap their foot on the ground at the furthest reach. Participants were given an approximately 1-min rest between trials.

The anterior, posteromedial, and posterolateral reaches were performed on each leg. The starting position was standing on one leg at the stance plate with the toes of the foot at the red line, and the other leg touching down lightly just behind the plate. The non-stance foot was reached out in the desired direction, pushing the reach indicator as far as possible while maintaining balance. The free foot had to be returned to the starting position under control. The participant might not touch down the free leg during the movement to keep balance or put their foot on top of the reach indicator to gain support and could not kick out the indicator. All measurements were taken from the red line on the stance plate, to the nearest .5 cm. The distance was read from the test device. Each test was repeated three times, and the maximum reach in each direction was recorded. The results were calculated taking limb length into consideration, to determine a “composite reach distance”. Lower-limb length was measured from the anterior superior iliac spine to the most distal part of the medial malleolus for each participant by using a tape measure while the participant lay in the supine position. Asymmetry has also been assessed by comparing the results from each leg.

#### 2.3.2 The biodex isokinetic leg muscle strength test

The strength of knee extensors and knee flexors was measured using Biodex System 3 (Biodex Corporation, Shirley, New York, United States). The isokinetic dynamometer provided constant angular velocity with accommodating resistance throughout a joint’s range of motion. Specific muscle groups of quadriceps and hamstring have been assessed for the following values: relative peak torque (peak torque per body weight), work (a functional value of muscle performance), and power (the muscle performance overtime). Selecting low strength speed (60°/s), medium fast speed (180°/s), and high endurance speed (300°/s) as isokinetic testing speeds was essential for optimal strength assessment in gymnasts who utilise both strength and fast speeds, as stated by Baltzopoulos and Brodie (1989).

The reliability of the Biodex’s concentric mode is high for the measurements of peak torque and single repetition work at speeds ranging from 60°/sec to 300°/sec (Feiring et al., 1990). The intraclass correlation coefficients (ICCs) of knee extension peak torque are .95 at 60°/s, .96 at 180°/s, .95 at 240°/s, and .97 at 300°/s. The ICCs of knee extension work are .96 at 60°/s, .97 at 180°/s, .96 at 240°/s, and .95 at 300°/s. The ICCs of knee flexion peak torque are .98 at 60°/s, .93 at 180°/s, .93 at 240°/s, and .82 at 300°/s. The ICCs of knee flexion work are .94 at 60°/s, .95 at 180°/s, .96 at 240°/s, and .93 at 300°/s.

Set-up and positioning were as follows: dynamometer orientation of 90°, dynamometer tilt of 0°, seat orientation of 90°, and seatback tilt of 70°–85°. Axis of rotation was through the lateral femoral condyle on a sagittal plane. Ready position was full flexion.

Prior to testing, participants were familiarised with the test using different random target angles. All measurements of the strength of knee joint extensors and flexors were performed from a sitting position with 90° average angle of the body and upper leg. Bands for stabilization were positioned over the body, hips, and distal part of upper leg for the leg that was tested. During the whole procedure of isokinetic testing examinees held their hands crossed on their chests. They started the test with the dominant lower limb**.** The test protocol consisted of a series of five repetitions at 60°/s, followed by 10 repetitions at 180°/s and 15 repetitions at 300°/s. All series were performed in the concentric/concentric mode for knee flexion/extension respectively, and with a 30 s interval between them. A resting time of 3 min was given between both leg measurements. As recommended by Drouin et al. (2004), the test examinees were given instructions that they had to give their maximum effort for each exercise. The peak torque values were recorded and analysed after being normalised by body weight for each leg and each angular speed. The hamstring/quadriceps ratio was calculated by dividing the concentric peak torque of hamstrings by that of quadriceps during the same contraction speed. Bilateral muscle strength difference was defined according to a previous study (Lockie et al., 2012).

### 2.4 Statistical analysis

(IBM SPSS Statistics for Windows, Version 23.0. Armonk, NY: IBM Corp). Data analysis was performed using the SPSS program for Windows, version 24.0 (IBM SPSS Statistics for Windows, Version 24.0. Armonk, NY: IBM Corp; 2016). The normality of data was analysed using the Shapiro-Wilk test. An alpha level of .05 was considered significant. Data was normally distributed and no significant differences in sample variance were detected.

Power analysis was performed with G*Power 3.1.9.2 software (Faul et al., 2009). Using the one-way ANOVA test, setting the alpha error at .05, the Power at .80, and comparing three groups with a large effect size (*f* = .40), we obtained a total sample of 24 participants. However, the total sample size in our study was 18, due to the fact that inclusion criteria required only elite competitive gymnasts.

Between-group comparisons were performed using one-way ANOVA with repeated measures. A Tukey *post hoc* test and Mann Whitney *U* test were used to identify the comparisons that were statistically significant. The significance level was set at *p <* .05. Data is expressed as mean ± standard deviation.

The relationship between values of dynamic balance and isokinetic leg muscle strength was determined by Pearson’s correlation coefficients. The level of significance was set at *p <* .05.

The magnitude of the effect size of the correlation was evaluated according to Cohen (1988). Cohen’s d was calculated with G*Power 3.1.9.2 software and interpreted as trivial (.00–.19), small (.20–.59), moderate (.60–1.19), large (1.20–1.99), and very large (>2.00) (Hopkins, 1997).

## 3 Results

### 3.1 A comparison of variables of dynamic balance and isokinetic leg muscle strength in rhythmic, artistic, and aerobic gymnasts

The composite score in the Y-balance test of the non-dominant and dominant symmetry differed significantly among groups of rhythmic, artistic, and aerobic gymnasts (Table 2). These between-group differences may be ascribed to their different demands on maintenance of balance under dynamic conditions within their sport specializations.

**TABLE 2:** Reach distances in anterior, posteromedial and posterolateral directions and the composite score in the Y-balance test in rhythmic, artistic and aerobic female gymnasts.

	Anterior D leg distance [cm]	Anterior ND distance [cm]
	Mean (SD)	Mean (SD)
Rhythmic gymnasts	58.50 (4.32)	60.00 (5.55)
Artistic gymnasts	69.40 (7.20)	67.80 (7.01)
Aerobic gymnasts	67.00 (5.42)	65.14 (6.54)
Between-group differences	*F* = 4.933, *p* = .023	*F* = 2.658, *p* = .103
	Posteromedial D leg distance [cm]	Posteromedial ND leg distance [cm]
	Mean (SD)	Mean (SD)
Rhythmic gymnasts	103.67 (4.50)	103.83 (8.95)
Artistic gymnasts	109.80 (1.92)	109.00 (6.40)
Aerobic gymnasts	106.57 (5.26)	104.86 (6.91)
Between-group differences	*F* = .944, p = .411	*F* = 1.503, p = .254
	Posterolateral D leg distance [cm]	Posterolateral ND leg distance [cm]
	Mean (SD)	Mean (SD)
Rhythmic gymnasts	100.83 (6.01)	100.83 (4.36)
Artistic gymnasts	103.20 (4.76)	103.80 (2.39)
Aerobic gymnasts	105.71 (6.73)	106.43 (5.38)
Between-group differences	*F* = 1.956, *p* = .176	*F* = 1.329, *p* = .294
	Composite score of the D symmetry [%]	Composite score of the ND symmetry [%]
	Mean (SD)	Mean (SD)
Rhythmic gymnasts	101.59 (5.46)	101.17 (4.47)
Artistic gymnasts	114.80 (2.22)	114.94 (2.55)
Aerobic gymnasts	106.43 (6.90)	106.32 (7.48)
Between-group differences	*F* = 3.536, *p* = .041	*F* = 4.804, *p* = .015

D, Dominant; ND, Non-dominant.

However, there were no significant differences in both posteromedial and posterolateral reach distances on both dominant and non-dominant leg and anterior non-dominant leg distance among these groups except for anterior dominant leg distance. Nevertheless, moderate to large effect sizes for dominant leg reach distances in anterior, posteromedial and posterolateral directions (1.734, .592, and .765 respectively) indicate that differences between rhythmic and aerobic gymnasts are significant from the practical point of view. Similar results were observed for differences between groups of rhythmic and artistic gymnasts in anterior, posteromedial and posterolateral directions (1.836, 1.772 and .437 respectively). However, only small effect sizes have been found for differences between artistic and aerobic groups in anterior, posteromedial and posterolateral directions (.377, .516, and .431 respectively). It seems that leg dominance does not play a role in dynamic balance among rhythmic, artistic and aerobic female gymnasts. Significant between-leg difference was only revealed for distance reached in anterior direction by the dominant leg in a group of rhythmic gymnasts (*F* = 5,944; *p* = 0.013). This may be also corroborated by small effect sizes ranging from .023 to .310 for reach distances in all three directions.

Furthermore, average power produced during knee extension and knee flexion at 60°/s, 180°/s and 300°/s differed significantly among these groups (Table 3). Similarly, total work was significantly different among these groups in almost all conditions, except for the non-dominant extension at 60°/s and the dominant extension at 180°/s (Table 4).

**TABLE 3:** Average (AVG) power produced during knee extension and knee flexion at 60°/s, 180°/s and 300°/s in rhythmic, artistic, and aerobic female gymnasts.

	AVG power during D leg extension at 60°/s [J]	AVG power during ND leg extension at 60°/s [J]	AVG power during D leg flexion at 60°/s [J]	AVG power during D leg flexion at 60°/s [J]
Rhythmic gymnasts	49.07 (19.91)	59.88 (17.96)	27.32 (10.26)	35.47 (8.50)
Artistic gymnasts	53.86 (10.12)	55.02 (13.35)	34.16 (10.40)	33.92 (8.13)
Aerobic gymnasts	80.20 (13.74)	76.36 (10.06)	47.47 (13.59)	47.71 (6.76)
Between-group differences	*F* = 7.76, p = .005	*F* = 4.07, p = .039	*F* = 4.97, p = .022	*F* = 6.03, p = .012
	AVG power during D leg extension at 180°/s [J]	AVG power during ND leg extension at 180°/s [J]	AVG power during D leg flexion at 180°/s [J]	AVG power during ND leg flexion at 180°/s [J]
Rhythmic gymnasts	102.63 (30.52)	109.23 (32.93)	63.15 (17.99)	58.78 (20.60)
Artistic gymnasts	104.02 (19.00)	97.40 (14.19)	66.64 (11.06)	61.82 (16.45)
Aerobic gymnasts	140.79 (17.84)	139.84 (18.65)	91.44 (13.74)	91.53 (10.31)
Between-group differences	*F* = 5.64, p = .015	*F* = 5.34, p = .018	*F* = 7.14, p = .007	*F* = 8.27, p = .004
	AVG power during D leg extension at 300°/s [J]	AVG power during ND leg extension at 300°/s [J]	AVG power during D leg flexion at 300°/s [J]	AVG power during ND leg flexion at 300°/s [J]
Rhythmic gymnasts	102.87 (17.07)	105.22 (32.92)	63.12 (15.28)	56.92 (22.29)
Artistic gymnasts	102.04 (8.71)	93.44 (13.17)	65.10 (12.56)	59.08 (8.87)
Aerobic gymnasts	133.44 (18.49)	138.36 (13.48)	83.54 (16.30)	84.66 (11.38)
Between-group differences	*F* = 8.07, p = .004	*F* = 6.99, p = .007	*F* = 3.63, p = .049	*F* = 6.45, p = .009

D, Dominant; ND, Non-dominant.

**TABLE 4:** Total work produced during knee extension and knee flexion at 60°/s, 180°/s and 300°/s in rhythmic, artistic, and aerobic female gymnasts.

	Total work during D leg extension at 60°/s	Total work during ND leg extension at 60°/s	Total work during D leg flexion at 60°/s	Total work during ND leg flexion at 60°/s
Rhythmic gymnasts	507.32 (203.04)	588.52 (182.37)	286.98 (98.60)	350.70 (78.86)
Artistic gymnasts	523.78 (95.17)	533.72 (148.09)	336.70 (105.27)	328.52 (87.19)
Aerobic gymnasts	763.46 (112.03)	741.64 (99.74)	458.19 (124.80)	463.77 (67.69)
Between-group differences	*F* = 6.26, *p* = .011	*F* = 3.44, *p* = .059	*F* = 5.12, *p* = .019	*F* = 5.58, *p* = .015
	Total work during D leg extension at 180°/s	Total work during ND leg extension at 180°/s	Total work during D leg flexion at 180°/s	Total work during ND leg flexion at 180°/s
Rhythmic gymnasts	790.68 (205.53)	832.23 (229.61)	511.90 (127.19)	491.83 (144.02)
Artistic gymnasts	773.72 (164.12)	733.92 (142.67)	523.42 (108.39)	496.24 (139.25)
Aerobic gymnasts	1014.11 (116.66)	1020.91 (125.14)	696.04 (72.46)	702.13 (63.65)
Between-group differences	*F* = .44, *p* = .654	*F* = 4.43, *p* = .031	*F* = 6.46, *p* = .009	*F* = 6.77, *p* = .008
	Total work during D leg extension at 300°/s	Total work during ND leg extension at 300°/s	Total work during D leg flexion at 300°/s	Total work during ND leg flexion at 300°/s
Rhythmic gymnasts	803.60 (128.45)	834.73 (229.57)	538.35 (111.32)	483.88 (167.91)
Artistic gymnasts	771.12 (77.62)	733.80 (130.05)	537.06 (107.48)	511.42 (87.23)
Aerobic gymnasts	993.49 (134.16)	1026.10 (116.64)	688.21 (100.42)	691.39 (77.59)
Between-group differences	*F* = 6.36, *p* = .01	*F* = 4.88, *p* = .023	*F* = 4.31, *p* = .033	*F* = 5.95, *p* = .013

D, Dominant; ND, Non-dominant.

However, there were no significant differences in isokinetic muscle strength during knee extension and knee flexion at any speed among rhythmic, artistic, and aerobic female gymnasts (Table 5). Angle of peak torque values are presented in Table 6. Nevertheless, there was a tendency for aerobic gymnasts to be stronger compared to artistic gymnasts and rhythmic gymnasts.

**TABLE 5:** Peak torque to weight ratio during knee extension and knee flexion at 60°/s, 180°/s and 300°/s in rhythmic, artistic, and aerobic female gymnasts.

	PT/BW during D leg extension at 60°/s [%]	PT/BW during ND leg extension at 60°/s [%]	PT/BW during D leg flexion at 60°/s [%]	PT/BW during ND leg flexion at 60°/s [%]
Rhythmic gymnasts	168.67 (49.00)	169.78 (47.72)	92.98 (18.08)	101.78 (6.78)
Artistic gymnasts	192.70 (36.26)	185.26 (62.80)	114.22 (25.91)	108.14 (30.31)
Aerobic gymnasts	220.56 (39.29)	210.43 (28.26)	118.41 (31.49)	119.11 (14.34)
Between-group differences	*F* = .622, *p* = .543	*F* = .323, *p* = .726	*F* = .149, *p* = .862	*F* = .519, *p* = .599
	PT/BW during D leg extension at 180°/s [%]	PT/BW during ND leg extension at 180°/s [%]	PT/BW during D leg flexion at 180°/s [%]	PT/BW during ND leg flexion at 180°/s [%]
Rhythmic gymnasts	122.65 (19.86)	112.87 (51.79)	78.97 (13.55)	77.27 (11.76)
Artistic gymnasts	143.08 (20.31)	135.72 (29.96)	93.62 (16.50)	86.88 (19.92)
Aerobic gymnasts	147.66 (16.22)	143.04 (14.62)	94.11 (12.36)	91.90 (9.01)
Between-group differences	*F* = .058, *p* = .943	*F* = 1.288, *p* = .289	*F* = .596, *p* = .557	*F* = .326, *p* = .724
	PT/BW during D leg extension at 300°/s [%]	PT/BW during ND leg extension at 300°/s [%]	PT/BW during D leg flexion at 300°/s [%]	PT/BW during ND leg flexion at 300°/s [%]
Rhythmic gymnasts	95.57 (11.39)	99.33 (10.15)	67.02 (13.15)	63.18 (15.60)
Artistic gymnasts	110.50 (8.36)	105.70 (11.67)	80.00 (8.56)	72.74 (11.03)
Aerobic gymnasts	111.60 (12.50)	110.63 (12.09)	75.81 (12.38)	76.66 (9.58)
Between-group differences	*F* = .251, *p* = .780	*F* = .995, *p* = .381	*F* = .040, *p* = .961	*F* = .062, *p* = .940

PT, Peak torque; BW, Body weight; D, Dominant; ND, Non-dominant.

**TABLE 6:** Angle of peak torque (APT) during knee extension and knee flexion at 60°/s, 180°/s and 300°/s in rhythmic, artistic, and aerobic female gymnasts.

	APT power during D leg extension at 60°/s [°]	APT power during ND leg extension at 60°/s [°]	APT power during D leg flexion at 60°/s [°]	APT power during ND leg flexion at 60°/s [°]
Rhythmic gymnasts	70.33 (7.09)	69.17 (7.19)	75.00 (7.51)	59.67 (20.13)
Artistic gymnasts	71.60 (7.23)	67.60 (6.02)	54.00 (22.05)	56.60 (23.90)
Aerobic gymnasts	71.43 (4.47)	68.43 (8.14)	58.57 (24.45)	41.29 (7.18)
Between-group differences	*F* = .0009, *p* = .999	*F* = .001, *p* = .999	*F* = .276, *p* = .760	*F* = .355, *p* = .704
	APT power during D leg extension at 180°/s [°]	APT power during ND leg extension at 180°/s [°]	APT power during D leg flexion at 180°/s [°]	APT power during ND leg flexion at 180°/s [°]
Rhythmic gymnasts	68.17 (6.11)	65.17 (7.76)	61.83 (13.79)	46.33 (25.84)
Artistic gymnasts	69.80 (8.47)	66.60 (9.02)	46.20 (9.47)	54.80 (12.03)
Aerobic gymnasts	73.29 (4.89)	72.43 (6.83)	58.57 (17.29)	61.00 (15.02)
Between-group differences	*F* = .016, *p* = .984	*F* = .037, *p* = .964	*F* = .189, *p* = .829	*F* = .178, *p* = .838
	APT power during D leg extension at 300°/s [°]	APT power during ND leg extension at 300°/s [°]	APT power during D leg flexion at 300°/s [°]	APT power during ND leg flexion at 300°/s [°]
Rhythmic gymnasts	77.83 (4.54)	70.17 (9.33)	50.50 (15.93)	45.17 (14.91)
Artistic gymnasts	76.00 (6.56)	77.40 (6.73)	42.60 (7.16)	43.40 (6.07)
Aerobic gymnasts	76.57 (4.69)	75.43 (5.38)	47.43 (14.15)	45.29 (14.44)
Between-group differences	*F* = .002, *p* = .998	*F* = .026, *p* = .974	*F* = .062, *p* = .940	*F* = .005, *p* = .995

D–Dominant, ND, Non-dominant.

Contralateral strength deficit expressed by agonist/antagonist ratio was, in all cases, under the critical value (62% for 60°/s, 76% for 180°/s, and 79% for 300°/s) for both the dominant and non-dominant leg at 60°/s (52.91% ± 8.65% and 57.06% ± 6.37%, respectively), 180°/s (64.19% ± 9.90% and 64.57% ± 5.91%, respectively), and 300°/s (67.83% ± 7.42% and 69.30% ± 6.23%, respectively) in aerobic gymnastics; at 60°/s (59.04% ± 4.54% and 60.04% ± 8.51, respectively), 180°/s (65.30% ± 5.04% and 64.22% ± 7.84%, respectively), and 300°/s (72.38% ± 4.39% and 68.88% ± 7.89%, respectively) in artistic gymnastics; and at 60°/s (56.92% ± 7.58% and 64.12% ± 18.62%, respectively), 180°/s (64.52% ± 6.97% and.60.43% ± 12.08%, respectively), and 300°/s (70.72% ± 15.00% and 63.75% ± 14.90%, respectively) in rhythmic gymnastics.

### 3.2 The relationship of reach distances in anterior, posteromedial, and posterolateral directions, as well as the composite score in the Y-balance test with an isokinetic muscle strength during knee extension and flexion at different velocities in female gymnasts of various disciplines

Results also showed a significant relationship between the composite score of the dominant limb symmetry and isokinetic dominant limb extension strength at 60°/s (*r* = .54), 180°/s (*r* = .87), and 300°/s (*r* = .84) in aerobic female gymnasts (Table 7). The composite score of the dominant limb symmetry was also associated with isokinetic dominant limb extension strength at 60°/s (*r* = .60), but not at 180°/s and 300°/s in artistic female gymnasts (Table 8). Similarly, there was a significant relationship between the composite score of the dominant limb symmetry and isokinetic dominant limb extension strength at 60°/s (*r* = .55), but not at 180°/s and 300°/s in rhythmic female gymnasts (Table 9).

**TABLE 7:** Relationships between reach distances in anterior, posteromedial and posterolateral directions and the composite score in the Y-balance test and isokinetic muscle strength during knee extension and knee flexion at 60°/s, 180°/s, and 300°/s in aerobic female gymnasts.

	The composite score	Anterior reach distance	Posteromedial reach distance	Posterolateral reach distance
Isokinetic D leg extension strength at 60°/s	.54	.46	.32	-.11
Isokinetic ND leg extension strength at 60°/s	.34	.29	.41	.30
Isokinetic D leg flexion strength at 60°/s	.34	.50	.39	.07
Isokinetic ND leg flexion strength at 60°/s	-.18	.64	.60	.71
Isokinetic D leg extension strength at 180°/s	.87	.26	.33	-.26
Isokinetic ND leg extension strength at 180°/s	.50	.03	.28	.37
Isokinetic D leg flexion strength at 180°/s	.20	.43	.54	.14
Isokinetic ND leg flexion strength at 180°/s	.19	.26	.20	.45
Isokinetic D leg extension strength at 300°/s	.84	.32	.60	-.01
Isokinetic ND leg extension strength at 300°/s	.59	.47	.57	.61
Isokinetic D leg flexion strength at 300°/s	.48	.14	.33	-.18
Isokinetic ND leg flexion strength at 300°/s	.39	.28	.10	.34

D–Dominant, ND, Non-dominant.

**TABLE 8:** Relationships between reach distances in anterior, posteromedial and posterolateral directions and the composite score in the Y-balance test and isokinetic muscle strength during knee extension and knee flexion at 60°/s, 180°/s, and 300°/s in artistic female gymnasts.

	The composite score	Anterior reach distance	Posteromedial reach distance	Posterolateral reach distance
Isokinetic D leg extension strength at 60°/s	.60	.23	−.55	.54
Isokinetic ND leg extension strength at 60°/s	.21	.18	.24	.12
Isokinetic D leg flexion strength at 60°/s	.48	.04	−.40	.49
Isokinetic ND leg flexion strength at 60°/s	−.05	.14	.31	.00
Isokinetic D leg extension strength at 180°/s	.17	.33	−.16	.46
Isokinetic ND leg extension strength at 180°/s	.37	.52	.56	.45
Isokinetic D leg flexion strength at 180°/s	.24	.20	−.15	.56
Isokinetic ND leg flexion strength at 180°/s	−.13	.26	.47	.07
Isokinetic D leg extension strength at 300°/s	.05	.36	−.08	.37
Isokinetic ND leg extension strength at 300°/s	.16	.27	.39	.20
Isokinetic D leg flexion strength at 300°/s	−.34	.28	.39	.41
Isokinetic ND leg flexion strength at 300°/s	.03	.02	.10	−.07

D–Dominant, ND, Non-dominant.

**TABLE 9:** Relationships between reach distances in anterior, posteromedial and posterolateral directions and the composite score in the Y-balance test and isokinetic muscle strength during knee extension and knee flexion at 60°/s, 180°/s, and 300°/s in rhythmic female gymnasts.

	The composite score	Anterior reach distance	Posteromedial reach distance	Posterolateral reach distance
Isokinetic D leg extension strength at 60°/s	.55	.14	−.25	−.34
Isokinetic ND leg extension strength at 60°/s	−.41	.52	.49	.61
Isokinetic D leg flexion strength at 60°/s	.59	.09	−.30	−.37
Isokinetic ND leg flexion strength at 60°/s	.02	.05	−.08	.15
Isokinetic D leg extension strength at 180°/s	.36	.31	.04	−.03
Isokinetic ND leg extension strength at 180°/s	−.49	.70	.47	.40
Isokinetic D leg flexion strength at 180°/s	.73	−.08	−.38	−-.09
Isokinetic ND leg flexion strength at 180°/s	.45	−.29	−.09	.14
Isokinetic D leg extension strength at 300°/s	−.25	.74	.41	.00
Isokinetic ND leg extension strength at 300°/s	−.34	.61	.42	.37
Isokinetic D leg flexion strength at 300°/s	.59	.00	−.20	−.10
Isokinetic ND leg flexion strength at 300°/s	−.03	.07	.41	.62

D, Dominant; ND, Non-dominant.

Balance and Muscle Strength in Gymnasts.

## 4 Discussion

Training in various gymnastic disciplines affects the development of balance positively and allows almost perfect stability, even in difficult conditions (Atilgan et al., 2012). Numerous investigations have compared athletes, artistic and acrobatic gymnasts, ballet or Latin dancers to non-athletes (Debu & Woollacott, 1988; Mesure and Cremieux, 1992; Era et al., 1996; Bardy and Laurent, 1998; Hain et al., 1999; Vuillerme et al., 2001; Asseman et al., 2004; Gerbino et al., 2007) in terms of their balance and postural stability. On the other hand, studies examining and comparing several gymnastic disciplines and lower limb strength, which also play a role in postural stability and performance, are relatively few in number.

Based on the previous knowledge, this study was designed to investigate possible differences of the selected parameters and the relationship between dynamic balance and isokinetic leg muscle strength in adolescent female gymnasts from various disciplines—rhythmic, artistic, and aerobic gymnastics. In order to succeed in sport, it is necessary to provide body control in a suitable mechanic as well as strength performance—that is, balance control should be improved (Sayers, 2000). The ability to provide and maintain balance is closely related to muscle tonus, muscle strength, muscle endurance, and joint movement flexibility (Howe et al., 2011; Leun et al., 2011; Ibiş et al., 2015). In addition, the lower extremity muscle group (ankle, hip, leg) is quite important in displaying balance skill (Runge et al., 2000; Deniskina and Levik, 2001). Knowledge of the relationship between dynamic balance and knee extensor/flexor could lead to the development of more effective balance interventions. To increase the performance of dynamic balance, athletes should practice exercises improving the isokinetic strength of knee circumference muscles (Aka and Altundag, 2020).

The current results showed a significant relationship between the composite score of the right symmetry and isokinetic right leg extension strength at 60°/s, 180°/s, and 300°/s only in aerobic gymnasts. The other finding is that the composite score of the right symmetry was also associated with isokinetic right leg extension strength at 60°/s, but not at 180°/s and 300°/s in artistic gymnastic group. Similarly, there was a significant relationship between the composite score of the right symmetry and isokinetic right leg extension strength at 60°/s, but not at 180°/s and 300°/s in rhythmic gymnasts.

### 4.1 The composite score in the Y-balance test of the left and right symmetry

In addition to the reach directions measurements, a composite score was calculated for Y-balance test. This parameter is important as gymnastics performance requires symmetrical, both-sides movements (FIG, 2021). Moreover, it is crucial to understand what role asymmetries in both functional movement and isolated strength play in injury risk or when returning to sport after injury. The results presented in this study demonstrate that the composite score in the Y-balance test of the left and right symmetry differed significantly among groups of rhythmic, artistic, and aerobic gymnasts. However, there were no significant differences in both posteromedial and posterolateral reach distances on both dominant and non-dominant leg and anterior non-dominant leg distance among these groups except for anterior dominant leg distance. Leg dominance is suggested to be important in rehabilitation and return to sport after knee injuries (van Melick et al., 2017). However, our findings are in accordance with other studies (Bahr & Holme, 2003; Michaelidis and Koumantakis, 2014; McGrath et al., 2016; Caetano Júnior, 2017) where the athletes also did not present a difference between dominant and non-dominant limb and that leg dominance had no influence on knee open kinetic chain proprioception and single-leg postural control (Alonso et al., 2011; Cug et al., 2016).

On the positive side, all three groups achieved a composite score over 95% which is associated with low risk of injury (Plisky et al., 2006; Schwiertz et al., 2020). Additionally, low values (94% and less) ​​in the composite score, as well as high differences between the reach of the limbs, predict a higher risk of injury in sports training. As shown in previous studies (Teyhen et al., 2014) there may be a risk of injury even with a high composite score, but large differences in the reach of the dominant and non-dominant limbs. Our results manifest there were no significant differences in anterior, posteromedial and posterolateral reach distances among groups of rhythmic, artistic, and aerobic gymnasts. No significant differences were found also in dominant and non-dominant limbs symmetry as compared with the established absolute side-to-side asymmetry injury risk cut-off value of greater than recommended values of 4 cm, 6 cm, and 6 cm in anterior, posteromedial and posterolateral differences, respectively (Plisky et al., 2006; Smith et al., 2015). However, additional factors contribute to performance on the YBT which, as intended by its creators, incorporates range of motion, closed chain stabilization, balance, and quadriceps and hamstring strength. Coaches should be aware of this when creating and planning the training.

### 4.2 Isokinetic muscle strength during knee extension and knee flexion at different speeds

Measures of isokinetic muscle strength are interpreted to represent dynamic muscle function and are the basis of athletes screening (Drouin et al., 2004). In addition, particular evaluation of knee flexor and extensor, as one of the largest and most important muscle groups, on a regular basis is suggested in terms of injury prevention. When measuring the isokinetic knee muscle strength, choosing angular velocities is important. Traditionally, slow speeds (60°/s to 120°/s) have been considered “strength speeds”, and medium to fast speeds (180°/s to 300°/s) have been considered “endurance speeds”. This is a fairly true assumption. It has been shown (Siqueira et al., 2002) that the differences in isokinetic muscle power between individuals at higher and lower levels of training advancement are greater at higher speeds of testing, since there are greater movement speeds, which mean quicker contractions.

It was expected that there would be differences in the power measured under isokinetic conditions between the gymnastic groups, as the type and intensity of physical activity are factors which—to a great extent—influence the manifestation of muscle power (Cormie et al., 2011). The current result obtained in average power, produced during knee extension and knee flexion at 60°/s, 180°/s, and 300°/s, significantly differed among rhythmic, artistic, and aerobic gymnasts. The highest values ​​were achieved by aerobic gymnasts, which is somewhat surprising. However, it could be explained by the character of performance of “intense aerobic movement patterns together with perfectly executed difficulty elements throughout the routine” (FIG, 2021).

Similarly, to previous findings, total work was significantly different among these groups in almost all conditions, except for the non-dominant limb extension at slow speed (60°/s) and the dominant limb extension at medium speed (180°/s). Such results signify practical significance of between-group differences resulting from different demands on lower limb strength in rhythmic, artistic, and aerobic gymnasts.

In the case of differences among the groups for variables of relative average isokinetic muscle power defined by peak torque/body weight; we can note that variables were not statistically relevant. Despite the fact knee extension and knee flexion at any speed did not differ among rhythmic, artistic, and aerobic female gymnasts, a slight tendency for aerobic gymnasts to be stronger compared to other two gymnastic group has been shown. These results can be explained either by training specificity, involving strength, resistance, balance, and flexibility, or by considering that body weight plays an important role in movement (Jaric, 2003; Edg et al., 2006). Aerobic gymnasts had greater body mass than the two other groups, and individuals with greater body mass manifest greater muscle strength—and thus, power. Previous studies have indicated that in tests of muscle power and strength evaluation, it is necessary to take body mass into account (Markovic and Jaric, 2005; Nedeljkovic et al., 2009; Toskić et al., 2020).

Increased dynamic loads during repetitive jumping, landing, and turning in gymnastics training and performance, as well as muscular imbalance between the hamstrings and quadriceps, may contribute to injury (Sweeney et al., 2018; Thomas and Thomas, 2019; Shapiro et al., 2020). The calculation of hamstring/quadriceps muscle strength ratio (H/Q ratio) between lower extremities, defined as contralateral deficits (Bamaç et al., 2008; Schons et al., 2018), is used to examine the functional abilities during speed-dependent movements, stability of the knee joint, and balance between the hamstring and quadriceps muscles (Bamaç et al., 2008; Cheung et al., 2012). A balanced muscle strength ratio between the agonist and antagonist muscle groups is extremely important for lower extremity stability and the prevention of knee injuries (Aagaard et al., 2000). Therefore, the determination of contralateral deficit and H/Q ratio in these athletes will play a key role in increasing performance and preventing injuries.

The magnitude of the hamstrings to quadriceps peak torque ratio (H/Q) may reflect the movement patterns during running or jumping (Rosene et al., 2001; Cheung et al., 2012), which are a frequent part in gymnastic routines. A typical isokinetic concentric H/Q for healthy athletes ranges from .50 to .80, depending on angular velocity, and indicates the muscle balance between the hamstrings and quadriceps (Hewet et al., 2008; Kong and Burns, 2010). Furthermore, the torque ratio of the agonist to antagonist knee muscles is associated with the demands of the sport, training adaptations, and level of competition (Rosene et al., 2001; Hewet et al., 2008; Kong and Burns, 2010; Cheung et al., 2012). Additionally, high and low performers demonstrated lower and higher extension torque deficits, respectively (Myers et al., 2018). Our gymnasts had values of H/Q ratio at 60°/s, 180°/s, and 300°/s for the dominant and non-dominant leg in the range described in the literature, and with increased values at faster speeds (Benell et al., 1998). According to Andrade et al. (2012), a H/Q ratio higher than 60% at 180°/s is a good marker, as only at high test speeds are the results similar to sports tasks. Our findings are in accordance with this, as the contralateral strength deficit expressed by agonist/antagonist ratio was in all cases bellow the critical value, with the lowest average of 60.43% ± 12.08% for non-dominant limb at 180% in rhythmic gymnastics group. By comparing data, no significant differences between extension and flexion of the same leg were found in any of the gymnastic group of adolescents. The results of this study showed that the gymnasts in all three groups had good performance of the lower limb muscles with balanced H/Q. However, a side-deficit has been demonstrated in individual cases, which could provide useful information for the prevention of injury and planning of strength training. Moreover, in a few cases, we noticed more than 10% asymmetry in relation to the norm. These findings are in an agreement with Yamamoto (1993), who demonstrated that the hamstring/quadriceps peak strength rate is influenced by angular speed rather than age, sex, and dominant and non-dominant features. As speed increases, the difference decreases. Such an imbalance between two muscle groups, particularly weak hamstring muscle, may cause injuries (Yamamoto, 1993).

In many sport trainings—gymnastics included—by performing the skills and difficulty elements, the knee extension muscle group is working more and the flexion muscle group is neglected. This state shows the result that the hamstring/quadriceps ratios in various athlete groups decrease more and may become an injury factor. While the hamstring/quadriceps rate shows muscular balance, it is also used as an indicator in preventing injuries. This indicates that near full extension, the hamstrings produce approximately 25%–30% of the moment that the quadriceps exert, supporting previous findings that the hamstring muscles play a significant role in providing dynamic joint stabilization during active knee extension (Dvir et al., 1989; Aagaard et al., 2000).

Regarding expertise and experience from gymnastics training, we can assume that volume of rebound jumps in various gymnastic training from a very young age may have an important, albeit different, influence on the dynamic and plyometric reliability of gymnasts. Gymnasts need to reach a high level of their rate of force development in an extremely short time, depending on disciplines. Aerobic gymnasts were found to be stronger compared to artistic gymnasts and rhythmic gymnasts. This result could be explained by the fact that performance in aerobic gymnastics, in contrast to artistic and rhythmic gymnastics, is realised mainly in standing, loading predominantly the lower part of the gymnasts’ body. In addition, the competitive routine must demonstrate continuous movements, which requires repeated dynamic rebounds (FIG, 2021). However, there were no significant differences in isokinetic muscle strength during knee extension and knee flexion at any speed among rhythmic, artistic, and aerobic female gymnasts. This may be ascribed to the small sample size of our groups.

Relationships between reach distances in anterior, posteromedial, and posterolateral directions and the composite score in the Y-balance test and isokinetic muscle strength during knee extension and knee flexion at 60°/s, 180°/s, and 300°/s.

The determination of the relationship between the risk of injury in gymnastics, knee flexion/extension contralateral deficit, hamstring/quadriceps ratio, and dynamic balance performance may play a role in increasing individual performance. In the literature, the relationship between knee muscle strength and jump height (Schons et al., 2018), risk of injury and knee muscle strength (Cheung et al., 2012; Kabacinski et al., 2018), and balance and injury risk (Clifton et al., 2013; Pourheydari et al., 2018) are clearly stated. However, the number of studies investigating the association between gymnasts’ isokinetic knee muscle strength and balance performance, when considering the specific group of adolescents and various gymnastic modalities, is insufficient.

The main goal of our investigation was to explore whether there is an association between the dynamic balance and isokinetic leg strength of female adolescent gymnasts in various disciplines. A significant relationship between the composite score of the dominant limb symmetry and isokinetic dominant limb extension strength at 60°/s, 180°/s, and 300°/s in aerobic female gymnasts was found. The composite score of the dominant limb symmetry was also associated with isokinetic dominant limb extension strength at the lowest angular velocity (60°/s), but not at “medium fast speed” (180°/s) and “endurance speed” (300°/s) in artistic female gymnasts. Similarly, there was a significant relationship between the composite score of the dominant limb symmetry and isokinetic dominant limb extension strength at 60°/s, but not at 180°/s and 300°/s in rhythmic female gymnasts. This may be ascribed to the specificity of performance in each gymnastic modality. While performance in aerobic gymnasts requires mainly repeated dynamic rebounds (jumps and leaps), artistic and rhythmic gymnasts activate almost equally their upper and lower body extremities during routines. These sport-specific variability in difficulty elements may explain differences in the relationship between dynamic balance and strength and fast speeds.

Therefore, the training should be focused on improvement of strength and/or fast speeds, as both significantly contribute to dynamic balance in adolescent gymnasts. As has been shown, a 10-week isokinetic training added to the traditional training improved the knee strength in preadolescent female gymnasts (Dallas et al., 2021). Although this leads to the improvement of aspects of the vault, it did not affect other technical aspects of the handspring performance. Thus, the determination of the knee joint muscles activation in young gymnasts can provide useful information for a better understanding of the mechanisms involved in strength production during athletes’ development.

Similar results to ours have been reported recently. In the study of Balci et al. (2020), weak and moderate relationships between balance skill and isokinetic knee muscle strength were determined in elite gymnasts. However, as previously reported, the knee joint muscle strength is related to balance ability, though the level of relationship may vary according to the type of strength and balance exercises, which might be also the case in our study. Śliwowski et al. (2021) showed the significant association between lower-limb muscle strength in the male soccer players and balance score, which could thus be identified as a significant predictor of balance control performance. Tasmektepligil (2016) reported a significant relationship between balance performance and isokinetic strength values at different angles for dominant and non-dominant legs among male soccer players. The author suggested to strengthen the muscles of players with isokinetic muscle strength exercise at specific degrees, as it is important for players in upgrading the static and dynamic balance values. A study by Soylu et al. (2020) reveals that the dynamic balance performance of the athletes increased as knee extension and flexion muscle strength increased, and demonstrated a negative significant correlation between the overall stability index (Biodex Balance System) with knee extension, and flexion peak torque of the dominant and non-dominant limb of angular velocity at 60°s^−1^ and 180°s^−1^ in female volleyball players. On the contrary, López-Valenciano et al. (2019) reported no significant correlation between concentric knee muscle strength and balance control. Findings of Tatlici et al. (2021) also showed that dynamic balance and leg muscle (hamstring and quadriceps) strength are independent of each other in the specific group of wrestlers.

### 4.3 Limitations of the study

The major limitation of the study is small sample size. The number of participants in each gymnastic group has been restricted, as the selection of only elite competitive level gymnasts was required. Therefore, recruiting a larger number of athletes at this level is constricted. We should also be aware of a certain genetic influence on the performance of high-level gymnasts (Maciejewska-Skrendo et al., 2021). Thus, because of these limitations, extrapolation of our finding into a general gymnastic should be performed with caution, and care should be taken when applying the study results. Another limitation is that only the muscle groups of quadriceps and hamstring, and only concentric knee flexion/extension, were evaluated by the isokinetic strength testing. Therefore, further research should focus on other muscle groups, as well as eccentric strength measurements, which are important for success in specific gymnastic performance. However, our study provides original data of female adolescent gymnasts in three different modalities, which can provide some basis for further research. It serves to the other investigations data on the possible relationship between dynamic balance and isokinetic leg muscle strength in these specific groups of athletes. Nevertheless, longitudinal studies are needed to investigate the possibility of the long-term effect of various gymnastics training on balance, strength, and other motor abilities.

## 5 Conclusion

There are significant differences in the composite score in the Y-balance test of the dominant and non-dominant symmetry in rhythmic, artistic, and aerobic gymnasts. Similarly, average power and total work differ significantly among these groups during isokinetic knee extension and knee flexion at almost all velocities used. The composite score of the right symmetry is associated with isokinetic right leg extension strength at 60°/s in aerobic, artistic, and rhythmic gymnasts, whereas at 180°/s and 300°/s in the aerobic group only. This relationship between dynamic balance in terms of maintenance of postural stability on a dominant leg and isokinetic muscle strength of the dominant leg most likely reflects sport-specific performance in female adolescent gymnasts. While specific elements in aerobic, artistic, and rhythmic gymnastics require both maintenance of dynamic balance in unstable positions and strength of lower limbs during various take-offs and landings, the performance in aerobic gymnastic additionally requires production of high force in a short amount of time. Therefore, their training should be focused on improvements of muscle strength and fast speeds, as both contribute significantly to dynamic balance in adolescent gymnasts.

## Data Availability

The raw data supporting the conclusion of this article will be made available by the authors, without undue reservation.
